# Effect of Passive Hyperthermia on Working Memory Resources during Simple and Complex Cognitive Tasks

**DOI:** 10.3389/fpsyg.2017.02290

**Published:** 2018-01-11

**Authors:** Nadia Gaoua, Christopher P. Herrera, Julien D. Périard, Farid El Massioui, Sebastien Racinais

**Affiliations:** ^1^School of Applied Sciences, London South Bank University, London, United Kingdom; ^2^Athlete Health and Performance Research Centre, Aspetar Orthopaedic and Sports Medicine Hospital, Doha, Qatar; ^3^Department of Kinesiology & Human Performance, Sul Ross State University, Alpine, TX, United States; ^4^Research Institute for Sport and Exercise, University of Canberra, Canberra, ACT, Australia; ^5^Cognition Humaine et Artificielle (CHArt), UFR de Psychologie, Université Paris 8, Paris, France

**Keywords:** EEG, hyperthermia, cognitive tasks, task complexity, overload

## Abstract

The aim of this study was to verify the hypothesis that hyperthermia represents a cognitive load limiting available resources for executing concurrent cognitive tasks. Electroencephalographic activity (EEG: alpha and theta power) was obtained in 10 hyperthermic participants in HOT (50°C, 50% RH) conditions and in a normothermic state in CON (25°C, 50% RH) conditions in counterbalanced order. In each trial, EEG was measured over the frontal lobe prior to task engagement (PRE) in each condition and during simple (One Touch Stockings of Cambridge, OTS-4) and complex (OTS-6) cognitive tasks. Core (39.5 ± 0.5 vs. 36.9 ± 0.2°C) and mean skin (39.06 ± 0.3 vs. 31.6 ± 0.6°C) temperatures were significantly higher in HOT than CON (*p* < 0.005). Theta power significantly increased with task demand (*p* = 0.017, η^2^ = 0.36) and was significantly higher in HOT than CON (*p* = 0.041, η^2^ = 0.39). The difference between HOT and CON was large (η^2^ = 0.40) and significant (*p* = 0.036) PRE, large (η^2^ = 0.20) but not significant (*p* = 0.17) during OTS-4, and disappeared during OTS-6 (*p* = 0.87, η^2^ = 0.00). Those changes in theta power suggest that hyperthermia may act as an additional cognitive load. However, this load disappeared during OTS-6 together with an impaired performance, suggesting a potential saturation of the available resources.

## Introduction

Exposure to heat stress leads to the development of hyperthermia when the prevailing ambient conditions become uncompensable. When hyperthermic, individuals stimulated with cold report feelings of pleasure, whereas displeasure is expressed when heat stress is further increased (Cabanac, 1987). Along with influencing the perception of pleasure, heat stress has been shown to influence cognitive function. Indeed, marked increases in core and/or skin temperature have been demonstrated to impair complex cognitive task performance (Hancock, 1986; Simmons et al., 2008). Recently, a hypothesis was developed linking this impairment to the alliesthesial change accompanying compensatory physiological responses to hot environmental conditions (i.e., strain related to thermoregulation) (Gaoua et al., 2012). More specifically, increases in temperature during heat exposure generated unpleasant stimuli, as measured by the Positive and Negative Affect Schedule (PANAS), which could be considered as a ‘cognitive load.’ It was proposed that this load might reduce the available resources for concurrent cognitive tasks. Interestingly, this could explain why reducing thermal discomfort, by cooling the head, for example, can restore some complex cognitive function in a hot environment (Gaoua et al., 2011b).

It has been suggested that performance of cognitive tasks under heat stress deteriorates when the total cognitive resources are insufficient for both the task and the thermal stress (Hocking et al., 2001). However, these findings have not been demonstrated empirically. Electroencephalography (EEG) measures recorded during cognitive tasks carried out in hot environments could provide insight into this process. Most EEG studies have focused on fluctuations in the theta (3–8 Hz) and alpha (8–12 Hz) power bands (Klimesch, 1999; Smith et al., 2001, 2004), as this allows discrimination between tasks having different workloads, under both simulated and actual working conditions (Wilson and Russell, 2003). Changes in alpha power are inversely related to cognitive processing with Lang et al. (1988) reporting decreased alpha activity when performing a concept formation task. Several other reports have also shown decreased alpha activity in association with increased task difficulty and the highest working memory loads during several cognitive tasks (Earle and Pikus, 1982; Gundel and Wilson, 1992). Conversely, an increase in theta power relative to rest has been reported during working memory (Gevins et al., 1997; Ishii et al., 1999; Mizuhara et al., 2004) and concentration tasks (Gevins et al., 1997; Ishii et al., 1999; Aftanas and Golocheikine, 2001; Jensen and Tesche, 2002). Such an increase in theta power over the frontal lobe is suggested to indicate an increase in the workload and demand on working memory (Kahana et al., 1999; Bastiaansen et al., 2002). Mean theta activity has also been shown to increase toward the end of difficult task sessions (Gevins et al., 1997) and when subjects are tired, but attempting to remain vigilant (Paus et al., 1997; Caldwell et al., 2003). An increase of theta power has also been reported at the frontal midline sites of the scalp during working memory and mental arithmetic tasks in the heat (Gevins et al., 1997; Ishii et al., 1999; Mizuhara et al., 2004). The elevated theta power is associated with an increase in concentration and heightened attention (Ishii et al., 1999; Aftanas and Golocheikine, 2001; Jensen and Tesche, 2002). This suggests that theta activity is not strictly related to the amount of information being manipulated, but to the level of mental effort being expended to cope with the task. As such, theta oscillations may be the best indicator of mental workload (Smith et al., 2001) and cognitive fatigue (Smith et al., 2004).

Therefore, the aim of this study was to determine whether cognitive resources are overloaded during passive hyperthermia by investigating the EEG responses to tasks of varying complexity. It was hypothesized that hyperthermia would represent a load and, as such, limit the resources available for performing cognitive tasks. This load would be characterized by a decrease in alpha activity and increase in theta activity under thermal strain, while an ‘overload’ during complex cognitive tasks would lead to an impairment in performance.

## Materials and Methods

### Participants

A total of 10 healthy males (35 ± 3 years, 79 ± 11 kg, 175 ± 5 cm; for age, weight, and height, respectively) volunteered for the study. Participants were asked to avoid all vigorous physical activity for the 24 h preceding the experiment. They were also asked to avoid caffeine and nicotine intake, as well as maintain their sleeping habits in the 24 h preceding each trial. The Institutional Human Ethics Committee approved the study, which was conducted in accordance with the Helsinki Declaration.

### General Procedure

After a familiarization trial, participants completed two experimental trials: one in a hyperthermic state in hot conditions (HOT: 50°C and 50% relative humidity) and another in a normothermic control state in temperate (CON: 25°C and 50% relative humidity) condition, separated by 4–7 days, in a counterbalanced design. Both experimental trials were conducted at the same time of day in an environmental chamber (Tescor, Warminster, PA, United States), with constant noise, light (212 lx), and ventilation (0.5–0.6 ms^-1^). During both trials, participants wore shorts and a T-shirt. In order to avoid the confounding effects of dehydration, water was provided *ad libitum* throughout both experimental trials.

### Familiarization Session

Commencing the experimental trials 1 week before, participants completed a familiarization session during which they performed the complete cognitive testing protocol and were accustomed to EEG procedures. In addition, the cognitive testing software (testing battery described below) provided a brief familiarization that was repeated before each test.

### Experimental Sessions

Before the experimental sessions, participants provided a urine sample for the measurement of urine-specific gravity (Pal-10-S, Vitech Scientific Ltd., West Sussex, United Kingdom) and were then weighed (nude body mass). After 20 min of rest for EEG electrode placement, they entered the environmental chamber. Participants initially walked for 10 min on a motorized treadmill (T170, COSMED, Rome, Italy) at 4 km.h^-1^. This procedure was done to minimize the initial decrease in core temperature (T_core_) related to the peripheral vasodilation. This protocol has been employed by previous studies to promote heat production without causing fatigue (Racinais et al., 2008). After walking, subjects sat resting in the upright position inside the environmental chamber for 35 min (CON) or until the target T_core_ of 39°C (HOT) was reached. This target T_core_ was selected based on previous studies showing decrements in cognitive performance from 38.7°C (Gaoua et al., 2011a) and to avoid subjects reaching too high temperatures by the end of the cognitive task. At this time, a planning task (OTS: One Touch Stockings of Cambridge) with on-going EEG recording was conducted. Prior to the cognitive testing, EEG recordings with eyes open were collected for 30 s in HOT or CON conditions.

### Temperature Recording

Core and skin temperatures were monitored using the VitalSense^®^ system (precision ± 0.01°C, Mini Mitter, Respironics, Herrsching, Germany). A wireless Jonah^TM^ ingestible thermometer pill, swallowed 5–7 h before the testing session, was used to measure T_core_. The validity of ingestible thermometer pills for monitoring T_core_ has been confirmed during both rest and exercise, making them a viable substitute for more invasive methods (Casa et al., 2005). Wireless XTP dermal adhesive temperature patches were used to measure chest (T_chest_), hand (T_hand_), and calf (T_calf_) skin temperatures. Both internal and external sensors sent data by telemetry to a single data logger every 60 s. Mean skin temperature (T_skin_) was calculated using Burton’s (1934) weighted formula: 0.5 T_chest_ + 0.14 T_hand_ + 0.36 T_calf_.

### Cognitive Testing

The OTS test was performed upon reaching 39°C in HOT or after 35 min of seated rest in CON. This test has been used in previous studies investigating the effect of hot environmental conditions on cognitive performance and was shown to be a valid tool to differentiate the effects of heat on simple and complex tasks (Gaoua et al., 2011a, 2012). This test was also used because instead of categorizing different tasks as simple and complex, it manipulates the complexity within the same task (Gaoua, 2010). Hence, the mechanism required to perform the task and the brain area being assessed remain constant, but the cognitive load required to successfully complete the task is manipulated.

The OTS was performed during each trial using Cantab software (CANTABeclipse, Cambridge Cognition, Cambridge, United Kingdom) and hardware (a tactile screen and a touch pad). Subjects were shown two displays containing three colored balls. The displays were presented in such a way that they could be perceived as stacks of colored balls held in stockings suspended from a beam. Along the bottom of the screen, there was a row of numbered boxes. Subjects were initially shown how to move the balls in the lower display to copy the pattern in the upper display. The experimenter completed one demonstration problem, where the solution required one move, following which the subjects completed three further practice problems, one each of two, three, and four moves before starting the test. For the test itself, subjects were shown further problems, requiring 2, 3, 4, 5, or 6 moves. Four of each of the task complexities was randomly presented to the participants. Participants had to mentally calculate the minimum number of moves required to solve the problems, and then to touch the corresponding box (1–6) at the bottom of the screen to indicate their response. The outcome measures were the number of problems solved on the first attempt, the latency to first responses (whether correct or wrong) and the latency to correct responses. Measures were analyzed for two different levels of complexity requiring either 4 (OTS-4, simple) or 6 moves (OTS-6, complex). Each measure was calculated by averaging the scores obtained over 4 trials.

### EEG Recordings

Continuous EEG data was recorded using the NicoletOne LTM system (Viasys Healthcare, Madison, WI, United States). Genuine gold cup electrodes (10 mm diameter, Grass Technologies, West Warwick, RI, United States) were affixed to the scalp with conductive paste (EC2, Grass Technologies, West Warwick, RI, United States) and secured with a small gauze pad. A lightweight hairnet was used to prevent the electrodes from moving. The primary recording electrode was placed at the Fz position and recorded with a paired mastoid reference and grounding electrode at the Fpz position according to the International 10–20 System (Homan et al., 1987). The frontal midline area has been shown to be a primary activity region within the brain during working memory tasks (Gevins et al., 1997; Ishii et al., 1999; Mizuhara et al., 2004). All electrode impedances were maintained below 10 kOhm. EEG data was sampled at 256 Hz, low pass filtered at 0.3 Hz, high pass filtered at 35 Hz, and stored on a computer hard disk for subsequent analysis. A Fast Fourier Transformation (FFT) was calculated with 2-s bins using a Hanning window with 75% overlap to yield the absolute power values for the theta (3–8 Hz) and alpha (8–12 Hz) frequency bands. These signal frequencies have been previously shown to have very high test–retest reliability when measured in the context of working memory tasks (McEvoy et al., 2000). Each measure was obtained by averaging the values from consecutively recorded 2-s data segments preceding correct responses during all OTS-4 and OTS-6 tasks. Digital markers were applied during data acquisition to represent the start and end (correct answer) of each task. When correct answers were given in less than 2 s, they were not used for analysis due to the limitations of the FFT analysis (i.e., at least 2 s of data were required for analysis). For the purpose of this study, measures at rest just before the cognitive tests (PRE) during the OTS-4 and the OTS-6 were analyzed.

### Thermal Perception

Thermal comfort and thermal sensation were recorded on visual analogic scales ranging from very comfortable (0) to very uncomfortable (20, white to black scale) and from very cold (0) to very hot (20, blue to red scale). The scores ranging from 0 to 20 were on the reverse sides of both scales and only visible to the researcher. Higher scores represented feeling less comfortable and hotter for thermal comfort and thermal sensation, respectively.

### Statistical Analysis

We used Shapiro–Wilk test and confirm that all data were normally distributed. Data were coded and analyzed in SPSS Version 17 software (SPSS, Chicago, IL, United States). A one-way within-subjects ANOVA was performed to study the effect of condition (CON, HOT). In addition, a two-way within-subjects ANOVA was performed to analyze the effect of condition as well as the effect of task (i.e., PRE, OTS-4, and OTS-6) and potential interaction on EEG data. All variables were tested using Mauchly’s procedure for sphericity. If a significant condition × task interaction was found, pairwise comparisons using a Bonferroni correction were used to compare the effect of condition at each time interval. The level of statistical significance was set at *p* < 0.05. Moreover, effect-sizes are described in terms of partial eta-squared (η^2^; with η^2^ ≥ 0.06 representing a moderate effect and η^2^ ≥ 0.14 a large effect, Cohen, 1969, pp. 278–280).

## Results

### Temperature and Thermal Perception

The T_core_ during the cognitive tasks was significantly higher in HOT (39.1 ± 0.3°C) than in CON (36.9 ± 0.2°C; *p* < 0.05, η^2^ = 0.97). T_skin_ was also significantly higher in HOT (39.5 ± 0.5°C) than in CON (31.6 ± 0.6°C; *p* < 0.05, η^2^ = 1). Participants reported a higher thermal sensation in HOT (16.2 ± 2.2) compared with CON (9.2 ± 1.5; *p* < 0.05, η^2^ = 0.95), as well as higher thermal discomfort in HOT (12.7 ± 5.3) relative to CON (5.8 ± 2.7; *p* < 0.05, η^2^ = 0.80). Body mass did not change from the start to the end of the CON trial (+0.1%; *p* > 0.05); however, a 0.4% body mass loss did occur during the HOT trial (*p* < 0.05). Urine-specific gravity prior to the experimental sessions was within the normal range for both HOT and CON (1.011 ± 0.007 vs. 1.016 ± 0.008 g/ml).

### Cognitive Function

**FIGURE 1:**
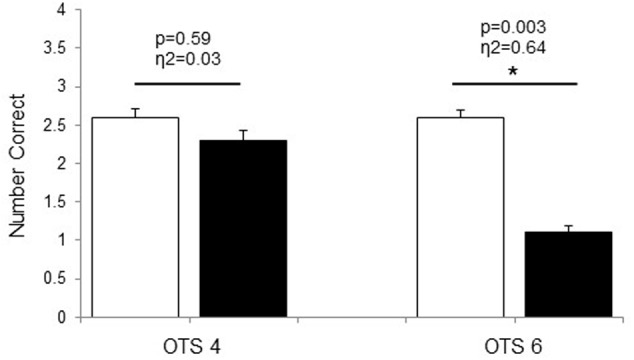
Number of problems solved on first choice during OTS-4 and OTS-6 in CON (white bars) and HOT (black bars) environments. Values are mean ± SEM. ^∗^Significant difference between HOT and CON conditions.

During the OTS-4, latency to first response was shorter in HOT than in CON (*p* = 0.018, η^2^ = 0.48; **Table 1**). There were no differences between conditions in the latency to the correct answer (*p* = 0.38, η^2^ = 0.09; **Table 1**) and the number of problems solved on first choice (i.e., accuracy) (*p* = 0.59, η^2^ = 0.03; **Figure 1**). For OTS-6, accuracy was significantly reduced in HOT compared with CON (*p* = 0.003, η^2^ = 0.64; **Figure 1**). The difference in the latency to the first response did not reach significance (*p* = 0.058), however, presented a large effect (η^2^ = 0.34; **Table 1**). Moreover, latency to the correct response was longer in HOT than in CON (*p* = 0.07, η^2^ = 0.57; **Table 1**).

**Table 1:** Latency to first choice and to the correct response for the OTS-4 and OTS-6 in CON and HOT presented in mean ± SEM.

		CON	HOT
OTS-4	Latency to first choice (s)	9.28 ± 2.56	7.10 ± 1.76^∗^
	Latency to correct (s)	10.13 ± 4.04	11.65 ± 3.52
OTS-6	Latency to first choice (s)	24.70 ± 14.27	15.93 ± 5.85
	Latency to correct (s)	21.92 ± 7.21	35.32 ± 9.72^∗^

*^∗^Significant difference between conditions, *p* < 0.05.*

### EEG Responses

**FIGURE 2:**
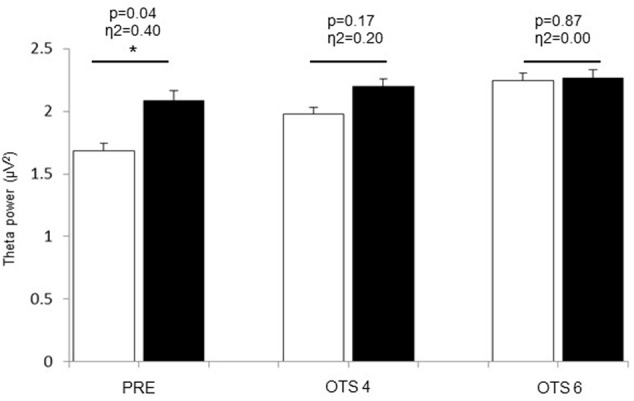
Theta power values obtained at PRE, during OTS-4 and OTS-6 in CON (white bars) and HOT (black bars) environments. Values are mean ± SEM. ^∗^Significant difference between HOT and CON conditions.

**FIGURE 3:**
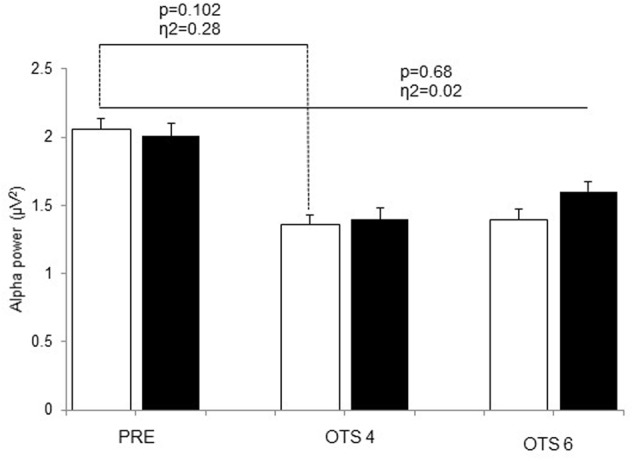
Alpha power values obtained at PRE, during OTS-4 and OTS-6 in CON (white bars) and HOT (black bars) environments. Values are mean ± SEM.

Theta power significantly increased with task demand (*p* = 0.017, η^2^ = 0.36) and was significantly higher in HOT than CON (*p* = 0.041, η^2^ = 0.39; **Figure 2**). The difference between HOT and CON was large (η^2^ = 0.40) and significant (*p* = 0.036) PRE, large (η^2^ = 0.20) but not significant (*p* = 0.17) during OTS-4, and disappeared during OTS-6 (*p* = 0.87, η^2^ = 0.00). Alpha power tended to decrease with task engagement with higher alpha power PRE (2.06 ± 0.8 μV^2^) compared to OTS-4 (1.4 ± 0.5 μV^2^; *p* = 0.102, η^2^ = 0.28; **Figure 3**), but did not further decrease with task complexity during the OTS-6 (1.4 ± 0.6 μV^2^). Changes in alpha power were not associated with T_core_ (*p* = 0.68, η^2^ = 0.02).

## Discussion

The aim of this study was to determine whether cognitive resources are overloaded during passive hyperthermia by investigating the EEG response (i.e., alpha and theta power) in the frontal lobe during simple and complex cognitive tasks. In accordance with previous studies, our data indicate that accuracy during complex cognitive tasks decreases in hot environments with and without an increase in T_core_ (Hancock, 1986; Racinais et al., 2008; Gaoua et al., 2011a,b). This decrease was previously associated with a dynamic change in T_core_ (Hancock, 1986; Gaoua et al., 2011b). Our study shows a similar decrease with a non-compensable but stable increase in T_core_ (≃39°C).

In the current study, hyperthermia was associated with a reduction in accuracy in the number of problems solved on first choice during the OTS-6 as well as an increase in the latency to the correct response (**Table 1**). For the first time, our data provide some EEG insight to explain these results. Indeed, EEG theta power was significantly elevated prior to task engagement (PRE, **Figure 2**), suggesting that hyperthermia imposed a cognitive load possibly related to the significant increase in thermal discomfort. Despite this, the simple task was successfully completed (**Figure 1**), but at a higher theta activity (OTS-4, **Figure 2**). However, it appears that theta power reached a threshold during the complex task beyond which it was not possible to allocate additional cognitive resources (OTS-6, **Figure 2**) to successfully complete the task, hence performance decreased (**Figure 1**).

It was previously suggested that theta power increases with greater memory demands (Gevins et al., 1997; Bastiaansen et al., 2002). The current results confirm that theta power significantly increases with task demand, as observed during the complex cognitive task in CON (**Figure 2**). However, the current data further shows that theta power also increases with hyperthermia. This increase in theta power could be related to the impact of physiological responses during heat stress on cognitive function. It is interesting to note that at this time subjects were hyperthermic but not actively engaged in any task (PRE). Accordingly, heat stress may represent a load that drains cognitive resources as in a dual task paradigm (Gaoua et al., 2011a,b).

Previous studies from Dubois et al. (1980, 1981) demonstrated a general slowing of EEG activity in clinical patients suffering from fever with a T_core_ of 38–40°C in association with an increase in theta power (Dubois et al., 1980, 1981). In the current study, the rise in T_core_ to ∼39°C induced an increase in theta power, which was higher both PRE and during the OTS-4 in HOT than in CON, despite there being no difference in performance. Similar results were observed in a study using steady-state visual evoked potentials (Hocking et al., 2001). This study demonstrated that with increasing T_core_, the potentials increased in amplitude and decreased in latency in the frontal lobe for working memory tasks and in occipito-parietal regions for vigilance tasks, with no significant difference in task performance compared to control conditions (Hocking et al., 2001). This indicates that despite changes in the underlying theta activity supporting task performance during hyperthermia, OTS-4 accuracy was not negatively impacted (**Figure 1**).

According to the multiple-resource theory, tasks using separate resources may be performed simultaneously without interference and, in the presence of resource conflict, the required resource can allocate part of its processing time to each task (Navon and Gopher, 1979). However, in the current experiment, hyperthermia was an ongoing factor during the cognitive task (i.e., concurrent processing time) and may have used similar cerebral resources as the cognitive task (i.e., frontal lobe resource conflict). This may have had an additive effect on cerebral resources in the area involved, rather than involving new brain areas (Adcock et al., 2000). The current data suggest that when performing a simple task in a hot environment (e.g., OTS-4), the cognitive load of the task and of the heat stress cumulate and lead to a higher load, as indicated by the higher theta values (**Figure 2**). Hence, working memory resources during the OTS-4 were increased to maintain task performance. In accordance with previous studies (Gaoua et al., 2011b), the speed of response to the first choice during the OTS 4 was higher in HOT compared to CON (**Table 1**) possibly in relation to an increase in nerve conduction velocity and in impulsivity, as previously observed in similar tasks performed in a hot environment (Racinais et al., 2008; Gaoua et al., 2011b). However, the latency to correct is a measure of both the time to process the information and the time to register the response on the screen. The absence of a difference between conditions in the latency to correct response may indicate that when hyperthermic, mental processing for a given task takes longer. The current data show that during the more complex OTS-6 task, speed of response was not different between conditions, but that more mistakes were made in HOT. This result is different from previous studies that have observed an improvement in reaction time during complex cognitive tasks in the heat (Simmons et al., 2008; Gaoua et al., 2012), and may relate to additional efforts being made to mobilize greater mental resources during the complex task. This premise is supported by the increase in theta power noted during OTS-6 compared to OTS-4 in CON.

Interestingly, our data showed that performance during the complex task (i.e., OTS-6) was impaired in HOT. This may be due to interference between two concurrent tasks requiring activation of the same part of the neural cortex (Klingberg, 1998). Indeed, interference has been observed between two cognitive tasks (Jaeggi et al., 2003), two motor tasks (Wenderoth et al., 2005), during the combination of a cognitive and a motor task in a temperate environment (Lorist et al., 2002), and during exercise-induced fatigue in a hot environment (Hocking et al., 2001). Our data suggest that heat stress also interferes with complex cognitive task performance and that cognitive resources may reach a critical threshold and become overloaded during hyperthermia, resulting in a decrease in performance. This supports the idea that there is a single pool of cognitive resources one can withdraw from (Kahneman, 1973) and that cognitive performance is impaired when combined with heat stress, but not when it is completed in normothermic conditions. In this case, the absence of a dual-task decrement during the OTS-4 can be explained by single resource theory on the assumption that the combination of tasks, or in the current study the combination of the OTS-4 and hyperthermia, does not exceed the upper threshold on the available resources (i.e., the task can be completed without interference) (Kahneman, 1973). It is worth noting that participants in the current study were passively exposed to heat stress with no option for behavioral thermoregulation, other than hydration. Hence, the decrement in resources could only influence the cognitive task (OTS-6).

Several reports have shown decreased alpha activity in the occipital and parietal regions of the brain in association with increased task difficulty and the highest working memory loads during several cognitive tasks (Earle and Pikus, 1982; Gundel and Wilson, 1992). Our study shows that this decrease in alpha activity also occurs in the frontal area with task engagement (OTS-4 and OTS-6, **Figure 3**). Higher alpha power is associated with reduced cortical activity and has been described as cortical idling, with a greater availability of resources for engagement in cognitive tasks (Klimesch, 1999). Interestingly, in the HOT condition alpha power appeared to be slightly higher during the OTS-6 than the OTS-4 (**Figure 3**). However, this task-related increase in alpha power during working memory tasks has been observed elsewhere (Jensen et al., 2002; Busch and Herrmann, 2003; Cooper et al., 2003; Sauseng et al., 2005, 2009). This paradoxical response in alpha activity during task engagement has been suggested to reflect the inhibition of task-irrelevant/interfering processes (Klimesch et al., 2011), such as the environmental and physiological heat stress in our experiment. Thus, we conclude that despite the attempt to manage the cognitive load associated with hyperthermia, there was no re-allocating of additional working memory resources as seen by no further increase in theta activity.

This study is not without limitations. Despite using a familiarization session and randomizing the trials in HOT and CON, it is possible that some other factors may have influenced cognitive performance and the associated EEG responses. These factors may include differences in motivation, fatigue, and arousal across trials. In addition, the sweat during HOT trial may have influenced the conduction of the electrodes and therefore EEG results. This would have been minimal as conductive paste was used to fix the electrodes to the scalp. Finally, only male participants were recruited for the study and the results may not be generalized to female populations. Future studies may consider including female participants to investigate gender differences. In fact, differences were previously suggested in a variety of psycho-behavioral and physiological factors including thermoregulatory responses and brain functions and structures that may influence the additional load imposed by hyperthermia.

In summary, the current data show that EEG theta power in the frontal area was significantly elevated PRE in HOT ambient conditions, suggesting that hyperthermia may in itself impose a cognitive load. Moreover, alpha power decreased during both simple and complex cognitive tasks. However, the simple task was successfully completed at the cost of an increase in mental load in the frontal area. Hence, during the complex task in hyperthermia, cognitive function may have reached a threshold beyond which it was not possible to allocate additional resources to successfully complete the cognitive task, and as a result performance declined.

## Ethics Statement

This study was carried out in accordance with the declaration of Helsinki and was approved by ‘Aspetar Orthopaedic and Sport Medicine Hospital Ethics Committee’ with written informed consent obtained from all subjects.

## Author Contributions

All authors have contributed to the manuscript. NG and SR developed the research design and protocol. NG, SR, and CH performed the experiment. NG prepared the first draft of the manuscript. JP critically reviewed it. All authors contributed to the final manuscript and gave final approval of the version to be published.

## Conflict of Interest Statement

The authors declare that the research was conducted in the absence of any commercial or financial relationships that could be construed as a potential conflict of interest.
